# Opioid crisis: addiction, overprescription, and insufficient primary prevention

**DOI:** 10.1016/j.lana.2023.100557

**Published:** 2023-07-12

**Authors:** The Lancet Regional Health – Americas

There are many sources of opioids, including raw poppy seeds, products made from poppy seeds (eg, poppy seed tea or kits for smoking), semisynthetic drugs (eg, heroin, morphine, and oxycodone), and synthetic drugs (eg, methadone and fentanyl). Over the centuries, opioids have been used as surgical analgesics, to stop diarrhoea and prevent tooth decay, as an adjuvant treatment of insomnia, to treat cancer and chronic pain, and as a recreational drug.

The opioid-abuse epidemic is a major problem in public health worldwide. In this special issue, researchers from the Americas present their views on the opioid crisis in Brazil, Mexico, and the USA. Although it is difficult to establish the major drivers of this crisis, experts point toward the influence by pharmaceutical companies, inadequate regulation, overprescribing by the medical profession, and increased use of illegal heroin and synthetic opioids. An explanation for the prevalent use or misuse of this class of drugs is their highly addictive power. To put it in context, heroin is in the most addictive drugs on the planet (category I, together with lysergic acid diethylamide and cannabis; among these drugs, only cannabis has a specific medical indication for cancer pain). Category II of most addictive drugs include commonly prescribed opioids for moderate and severe pain (hydrocodone, methadone, meperidine, oxycodone, and fentanyl). As a result, 60,000,000 people struggle with the addictive effects of opioids globally and more than 100,000 people die every year of opioid overdose, many of them with fentanyl, an analgesic drug that is 50–100 times more potent than heroin or morphine.

In the Americas, the opioid crisis has particularly hit the USA and Canada, affecting mostly young and middle-aged adults. In 2019, opioids were responsible for 15.8 and 6.4 deaths per 100,000 people in the USA and Canada, respectively. This emergency prompted the launch of the Stanford-Lancet Commission in 2022, which points out that the North American opioid crisis originated from a multi-system regulatory failure. Concerns have also been expressed in other territories in the region. For example, Guyana, Bolivia, and the Dominican Republic have the highest death rates due to opioid use after the USA and Canada, although these are much lower (<2 per 100,000 people). As in North America, fentanyl use is a growing problem in several Latin American countries. In Brazil, fentanyl is competing with codeine and oxycodone, which remain the main drivers of opioid use and abuse. Unfortunately, little is known about the status of fentanyl use in other countries in the Americas and high-quality research is urgently needed to better understand the real burden of opioid abuse in these populations.

Several measures have been proposed by the Stanford-Lancet Commission to tackle the opioid crisis, including better drug regulation, education (including the safe disposal of opioids and awareness of harmful effects), restrictions on opioid prescription, and promotion of prevention campaigns, for example the need for creating healthy environments as a strategy to reduce opioid addiction. Alarming statistics suggest that opioid overprescription has been a key factor in the US opioid crisis. Opioid addiction is also a big problem among physicians themselves. Nevertheless, despite a consistent decline in opioid prescription in the USA since 2013, the number of deaths continues to increase, indicating that other causes have become equally or more important, such as the incursion and popularity of synthetic opioids obtained from the illegal market and clandestine laboratories.

Naloxone, an opioid receptor antagonist, is the most widely used drug to treat opioid overdose, with the potential of preventing deaths. On March 29, 2023, the US Food and Drug Administration approved naloxone as a nasal spray to be classified as an over-the-counter drug. Some scientists have expressed optimism that this policy could increase the availability of naloxone and reduce stigma associated with the purchase of naloxone in pharmacies. However, no study has assessed the effectiveness of this policy, despite that over-the-counter naloxone has been available for many years in some countries, including Canada and Italy, the latter having implemented this policy more than 25 years ago. It is still premature to call this measure a positive policy as there are also concerns about affordability.

Despite being an opioid itself, methadone is used to treat opioid addiction, including during pregnancy, and to prevent severe withdrawal symptoms caused by quitting heroin and other opioids. However, in Mexico, the closure of the only methadone manufacturing plant in the country is threatening a shortage of this drug. Justified or not, this decision could have immediate consequences not only to opioid users but also to the whole society. In this issue, Bejarano and colleagues presented an overview of the opioid crisis in Mexico, the associated deaths linked to fentanyl, and the unresolved issues that aggravate this crisis, including the insufficient surveillance and emergency response, ineffective educational campaigns, unaccredited rehabilitation centres, poor availability of methadone, scarcity of naloxone, illegal market and production of fentanyl, and overall failure of adequate health policies.

Although commendable, most government efforts have focused mainly on secondary and tertiary prevention to avert relapses, overdoses, and deaths. Unfortunately, public health authorities and policy makers are still reluctant to prioritise primary prevention to tackle drug use and the opioid crisis. If we do not put enough focus on primary prevention to discourage people from using opioids as recreational drugs and from abusing medicated opioids, we will not be able to defeat the growing opioid crisis in our region and worldwide. More primary prevention campaigns must be implemented alongside currently favoured health policies.

